# Behaviour change techniques in personalised care planning for older people: a systematic review

**DOI:** 10.3399/bjgp20X714017

**Published:** 2021-01-26

**Authors:** Sadia Ahmed, Anne Heaven, Rebecca Lawton, Gregg Rawlings, Claire Sloan, Andrew Clegg

**Affiliations:** Leeds Institute of Clinical Trials Research, Clinical Trials Research Unit, University of Leeds, Leeds.; Academic Unit for Ageing and Stroke Research, University of Leeds, Bradford Institute for Health Research, Bradford Royal Infirmary, Bradford.; Psychology of Healthcare, School of Psychology, University of Leeds, Leeds; director, NIHR Yorkshire and Humber Patient Safety Translational Research Centre, Bradford.; School of Clinical Psychology, University of Sheffield, Sheffield.; MODS/BASIL Programme Mental Health and Addiction Research Group, Department of Health Sciences, Faculty of Sciences, University of York, York.; University of Leeds, Leeds; honorary consultant geriatrician, Bradford Royal Infirmary, Bradford; theme lead, NIHR ARC Yorkshire & Humber Improving Care for Older People with Frailty theme, Bradford.

**Keywords:** behaviour change techniques, older people, personalised care planning, quality of life, randomised controlled trial, systematic review

## Abstract

**Background:**

Personalised care planning (PCP) interventions have the potential to provide better outcomes for older people and are a key focus in primary care practice. Behaviour change techniques (BCTs) can maximise effectiveness of such interventions, but it is uncertain which BCTs are most appropriate in PCP for older adults.

**Aim:**

To identify BCTs used in successful PCP interventions for older people aged ≥65 years.

**Design and setting:**

Systematic review.

**Method:**

The authors searched 12 databases from date of inception to 30 September 2017. They identified randomised controlled trials (RCTs) of interventions involving participants aged ≥65 years, and contextually related to PCP. Five areas of risk of bias were assessed. The Michie *et al,* BCT taxonomy was used for coding.

**Results:**

Twenty-three RCTs involving 6489 participants (average age 74 years) described PCP interventions targeting the general older adult population and older people with specific long-term conditions (for example, heart disease, diabetes, stroke). Just over half of the studies were deemed to be at a low risk of bias. Eleven ‘promising’ BCTs were identified in five trials reporting significant improvements in quality of life (QoL). Six BCTs were reported in all five of these trials: ‘goal setting’, ‘action planning’, ‘problem solving’, ‘social support’, ‘instructions on how to perform a behaviour’, and ‘information on health consequences’. Modes of delivery varied.

**Conclusion:**

Future PCP interventions to improve QoL for people aged ≥65 years may benefit from focusing on six specific BCTs. Better reporting of BCTs would enhance future design and implementation of such interventions.

## INTRODUCTION

Personalised care planning (PCP), defined as *‘Explicitly engaging patients in a shared decision-making process involving both goal setting and action planning’*,^1^ embodies core principles of ‘person centredness’ and ‘shared decision making’ embedded in the NHS for the past 20 years.^2^^–^^6^ The aim of PCP is to support individuals to self-manage their own health and wellbeing, typically using behaviour change techniques (BCTs) to help achieve collaborative outcomes. The PCP process enables linkage to additional mechanisms for improving outcomes, including improved care coordination and better access to community resources.^1^^,^^7^ Recognised key outcomes of PCP are improved physical and mental health, self-management capabilities, health-related behaviours, and changes in health service use.^1^^,^^7^

**Figure 1.**
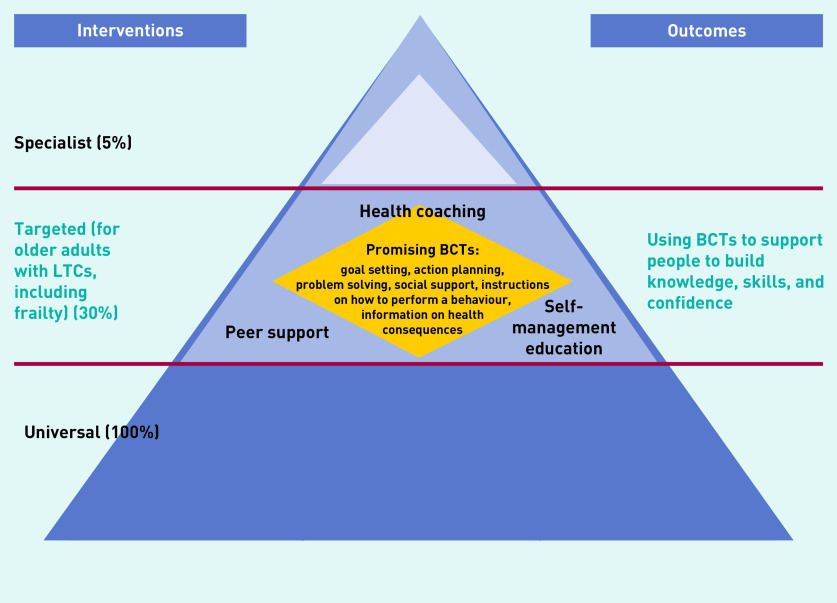
***Behaviour change techniques (BCT) within the Comprehensive Personalised Care model.*** ***LTCs = long-term conditions.***

In the UK, the publication in 2018 of the *Comprehensive Model of Personalised Care* consolidated evidence demonstrating PCP as a promising approach to achieve change (Figure 1).^7^ This informed the 2019 *NHS Long Term Plan*, and the linked work programme to implement personalised care nationally.^8^ As part of the *NHS Long Term Plan*, the NHS England Ageing Well programme specifies a multidisciplinary team approach to care for older people (generally defined as ≥65 years) defined as anticipatory care. Both personalised and anticipatory care were included in the draft 2020 primary care network direct enhanced service (PCN DES) specifications, but implementation was paused after the initial consultation period.^9^ Personalised care plans for people eligible for anticipatory care — for example, those with frailty — establish linkage across the individual specifications that are expected to form part of future GP contract negotiations.

The pause in implementation of the anticipatory and personalised care elements of the PCN DES allows reflection on how PCP services could be optimally designed for older people. Use of BCTs to help support development of self-management capabilities is recognised as central to successful PCP. BCTs can maximise intervention effectiveness by helping individuals achieve and sustain behaviour change,^10^ but the effectiveness of particular BCTs may vary across the life course, and it is currently unclear which BCTs are most relevant and effective in PCP for older people.

**Table:** How this fits in

Wider implementation of personalised care planning (PCP) is included in national policy in the linked NHS England Personalised Care and Ageing Well programmes, and is expected to be included in the 2021–2022 GP contract negotiations. Behaviour change techniques (BCTs) are central to implementation of PCP, but are contextual, and not all BCTs are appropriate for use with older people (aged ≥65 years). Building on the current policy and operational focus on implementation of PCP for older people in primary care, this review supports the targeted intervention component of the comprehensive personalised care model by identifying six specific BCTs that have been successfully used in interventions to improve quality of life for older people.

The Michie *et al* BCT taxonomy^11^ identifies 93 BCTs, enabling accurate identification and replication of intervention components and classification, and extraction of BCTs for the purpose of systematic reviews.^12^ The taxonomy has been used to identify the BCTs most prevalent and effective for various population groups and behaviours, and can help to develop interventions with a particular set of theoretical determinants underpinning behaviour.^13^ BCTs, used alone or in combination, map onto nine intervention functions (IFs) acting as broader mechanisms for change. Identifying IFs alongside BCTs indicates which behavioural change mechanisms might work best. Healthcare and allied professionals are increasingly trained in and use BCTs and IFs to inform practice and interactions with clients.^13^

The aim of this review was to identify relevant BCTs for use with older people to inform the development of the Personalised Care Planning for Older People with Frailty (PROSPER) intervention as part of a National Institute for Health Research Programme Grant for Applied Research (NIHR PGfAR).^14^

The objectives were to:

- systematically identify and describe randomised controlled trials (RCTs) evaluating PCP in older people (with or without frailty), examining health, behaviour, and quality of life (QoL) outcomes;
- identify behaviour change elements in these studies, exploring the potential effectiveness of BCTs in improving QoL outcomes for older people in the context of PCP.

## METHOD

### Search strategy

This review followed PRISMA guidelines.^15^ A systematic search was implemented in MEDLINE, PsycINFO, EMBASE, CINAHL, AMED, PubMed, Scopus, Applied Social Science Index, British Nursing Index, Health Technology Assessment, Cochrane Central Register of Controlled Trials, and the Cochrane Database of Systematic Reviews. Databases were searched from date of inception to 30 September 2017. Search terms were developed in collaboration with an information specialist.

### Eligibility criteria

This review focused on randomised controlled trials (RCTs) and cluster RCTs of interventions contextually related to PCP, that is, including ‘goal setting’ and ‘action planning’.^1^ Interventions had to:

- focus on one-to-one PCP (not group education);
- incorporate active involvement of the patients in a collaborative or shared decision-making process;
- include collaborative goal setting and action planning;
- include patient-based outcomes, for example, QoL and self-efficacy;
- encourage patients to set their own goals or priorities, and offer choices; and
- actively involve patients in planning treatment or care.

Studies had to include participants aged ≥65 years, or ≥50 years if the sample mean age was ≥65 years. Settings could include care homes, the community, and inpatient units.

### Screening

All reviewers screened the first 40 titles and abstracts from retrieved articles to ensure consistency in applying inclusion and exclusion criteria. The remainder of the articles were divided equally between the four reviewers. Following this, full texts of potentially eligible relevant articles were obtained. Full texts were divided equally between five reviewers, then screened, and reasons for exclusion recorded. Papers initially selected for inclusion were screened again and consensus was reached for the final list.

### Data extraction

Four reviewers extracted data, and 10% of the extractions were double-checked by another member of the team. Three reviewers with BCT taxonomy coding training^12^ independently coded all BCTs explicitly reported in both intervention and control conditions. It was important to note if specific/different BCTs were being used in the controls or as part of usual care as they may also affect outcomes. Table 1 shows the frequency of ‘promising BCTs’ in both intervention and control arms.

**Table 1.** Promising behaviour change techniques

**Behaviour change technique**	**Example**	**Interventions, *n***	**Controls, *n****^a^*
**Action planning**	Agreeing to eat three light meals a day, with at least one hot meal. Action planning needs to include thought about when, where, and how the behaviour will take place	23	0
**Goal setting (outcome)**	Goals generally need to be specific, measurable, achievable, relevant, and time bound (SMART), and the result of shared decisions. For example, getting to the shops and home without assistance	21	0
**Social support (unspecified)**	Getting a ‘blue badge’ to allow the person to go to the shops alone	21	2
**Problem solving**	Working to identify the barriers preventing individuals from engaging in behaviours and identifying ‘enablers’. For example, not being able to get to a social group because of lack of transport; problem solving should address how they might access the group	19	0
**Information on health consequences**	General education: information about the benefits of drinking enough water (hydration), or the negative effects of consumption of sugary foods if they have diabetes	19	6
**Credible source**	Using information from a well-known and respected source, for example, British Heart Foundation chair-based exercises	15	4
**Pharmacological support**	Using pharmacological support, including appetite stimulation, to improve the appetite in patients with weight loss	14	4
**Instructions on how to perform a behaviour**	Advising how to use online services from the local council	13	1
**Verbal persuasion about capability**	Focusing on an individual’s abilities and assets, and providing verbal encouragement	12	0
**Review outcome goals**	Checking if goals have been achieved, and exploring barriers to achievement	9	0
**Biofeedback**	Breathing exercises and monitoring with spirometer	4	0

a The control groups did not receive the intervention being trialled but in some cases did include BCTs as part of their ‘usual care’ or as a minor ‘add on’ to usual care. BCTs = behaviour change techniques.

Coding was reviewed by the whole team, and disparities resolved by consensus. Two reviewers replicated the process to code IFs. In an attempt to capture all relevant BCTs, the authors coded those that were definitely (++) and probably (+) present.^2^ They used BCT domain headings as codes when there was a lack of information to specify a technique. One of the authors who is a behaviour change expert provided advice and input on coding.

Using the Brown *et al* approach,^16^ the authors defined a BCT as ‘promising’ based on frequency (included in ≥25% interventions) and being present in at least two effective interventions, that is, those that reported statistically significant differences (*P*<0.05) at the latest available follow-up.

### Risk of bias assessment

Each reviewer assessed five areas of risk of bias using the Cochrane Collaboration Tool.^17^ Each entry was rated as low, high, or unclear risk, with 10% of assessments double-checked by a second reviewer and disagreements resolved by consensus. This assessment was not used to exclude studies or weight the findings, but to highlight where systematic error may have occurred.

### Data analysis

Findings were summarised using a narrative approach.

## RESULTS

### Literature search

**Figure 2.**
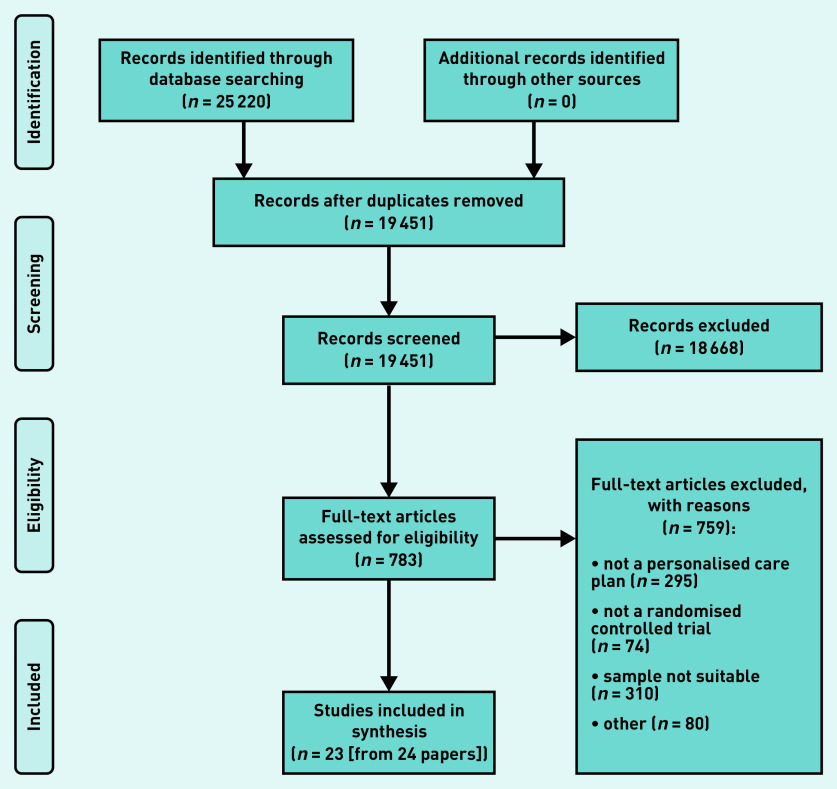
***PRISMA flow diagram.***

The search identified 19 451 unique articles. Following title/abstract screening, 783 full-text articles were assessed for eligibility, of which 759 were excluded. Twenty-three interventions reported in 24 articles met the criteria for inclusion (Figure 2).

### Study characteristics

There were 6489 participants (mean age 74 years) across 23 studies. Eleven of the 23 studies were conducted in the US and Canada,^18^^–^^28^ eight in Asia,^29^^–^^36^ two in Europe,^37^^,^^38^ and two in the UK.^39^^,^^40^ Most participants were female. Eleven of the studies focused on general older adult populations, six on participants with heart disease or angina,^29^^,^^30^^,^^36^^,^^38^^–^^40^ two on those with diabetes,^23^^,^^31^ two on stroke survivors,^21^^,^^37^ and two on nursing home residents.^34^^,^^35^ Five studies focused specifically on older people with Medicare insurance.^22^^,^^24^^,^^25^^,^^27^^,^^28^ Two studies focused specifically on older people with frailty, although frailty was not defined.^18^^,^^20^ Although more than half the trials explicitly mentioned behaviour change theory, details varied widely.

Delivery settings included participants’ homes (*n* = 9),^18^^–^^24^^,^^27^^,^^28^ hospitals (*n* = 5),^26^^,^^29^^,^^30^^,^^33^^,^^38^ primary care practices (*n* = 4),^24^^,^^25^^,^^32^^,^^39^ and a nursing home (*n* = 1).^34^ Modes of intervention delivery included online, via telephone, and face-to-face. Eleven interventions^18^^–^^20^^,^^24^^,^^25^^,^^27^^–^^30^^,^^36^^,^^38^ used both face-to-face and telephone delivery. Almost half of the interventions (10/23) were delivered by nurses.^18^^,^^24^^,^^25^^,^^28^^–^^30^^,^^32^^,^^38^^–^^40^ Other delivery agents were GPs,^39^ occupational therapists,^37^ volunteers,^20^ and researchers.^36^

The majority of interventions aimed to improve self-care or self-management of a disease. Others aimed to improve participants’ independence in their homes;^21^^,^^37^ ability to carry out activities of daily living,^37^ or engagement in therapy.^26^ Some also aimed to reduce use of health services.^24^ Supplementary Table S1 contains detailed information on study characteristics.

### Risk of bias assessment

Eleven of 23 studies scored low on the majority of the criteria for risk of bias.^18^^,^^20^^,^^23^^,^^26^^,^^27^^,^^29^^–^^31^^,^^33^^–^^37^ Generally, there was insufficient information on method of randomisation, allocation concealment, and blinding, but a high risk of bias was observed on ‘blinding of participants and personnel’.^17^ Only one study^18^ had a green risk assessment in this area. All other studies were either amber or red. The red, amber, and green assessment of risk for each of the criterion is shown in Supplementary Table S2.

### Findings

Seventeen of the 23 studies reported statistically significant findings on one or more outcome measure between groups^23^^,^^30^^,^^33^^,^^35^ or a within-group difference over time.^31^ There were significant findings relating to mortality and disease-specific outcomes in five studies.^2^^,^^27^^,^^30^^,^^31^^,^^36^ Five studies demonstrated significant improvements in mental health outcomes.^23^^,^^32^^,^^34^^,^^35^^,^^40^ Five also showed significant improvements in behavioural outcomes such as physical activity or attendance at fitness classes,^25^^,^^26^^,^^28^^,^^29^^,^^33^ and five for QoL outcomes.^23^^,^^30^^,^^31^^,^^33^^,^^35^

### Behaviour change techniques

#### Intervention groups

Forty-seven of the 93 BCTs in the taxonomy were reported in the intervention groups. Supplementary Table S3 summarises the BCTs used in intervention and control groups.

#### Control groups

Twelve BCTs were identified in the control groups, with the most common ones being ‘social support’ (practical), ‘information about health consequences’, ‘credible source’, and ‘pharmacological support’.

#### Intervention functions

Six of the nine IFs were coded. Table 2 shows ‘persuasion’ coded for all interventions. ‘Enablement’ and ‘education’ were also prevalent. Most studies satisfied more than one IF. The mean number of IFs per study was three. ‘Incentivisation’, ‘coercion’, and ‘restriction’ were not coded. Many interventions provided some form of lifestyle information or education. For more detailed intervention descriptions, including significance, see Supplementary Table S4.

**Table 2.** Frequency of intervention functions

**Intervention function**	**Frequency of use, *n***
Persuasion	23^18^^–^^40^
Enablement	17^18^^,^^19^^,^^21^^–^^32^^,^^38^^,^^40^
Education	17^8^^,^^20^^,^^21^^,^^23^^,^^25^^,^^27^^,^^29^^–^^36^^,^^38^^,^^39^^,^^40^
Training	9^18^^,^^24^^,^^25^^,^^27^^,^^28^^,^^31^^,^^33^^,^^37^^,^^40^
Environmental restructuring	6^23^^,^^24^^,^^26^^–^^28^^,^^37^
Modelling	4^20^^,^^28^^,^^33^^,^^36^

#### Promising behaviour change techniques in trials reporting quality of life outcomes

There were 11 trials that included QoL as an outcome. Of these, five trials reported significant improvements for QoL, either between groups*,*^23^^,^^30^^,^^31^^,^^33^^,^^35^ or within groups over time.^31^ One of the significant QoL interventions was web based.^23^ From these five trials, the authors identified 11 ‘promising’ BCTs. Of the 11 promising BCTs, six were present in all five of the trials. These were: ‘goal setting’, ‘action planning’, ‘problem solving’, ‘social support’, ‘instructions on how to perform a behaviour’, and ‘information on health consequences’. ‘Goal setting’, divided into ‘goal setting (behaviour)’ and ‘goal setting (outcome)’, aligned with the BCT taxonomy. ‘Unspecified social support’, usually including advice, encouragement, or coaching, was also a promising BCT; emotional social support was rarely identified. Examples of all 11 BCTs are shown in Table 2.

## DISCUSSION

### Summary

The authors identified 23 trials involving 6489 participants that used BCTs in the context of PCP interventions with older people. Interventions differed in terms of setting, mode of delivery, intervention provider, and reported outcomes.

Six of eleven promising BCTs were identified in five studies that showed improvements in QoL. Goal setting (behaviour) and goal setting (outcome) featured along with action planning, problem solving, social support, instructions on how to perform a behaviour, and information on health consequences. ‘Emotional’ social support was rarely mentioned, but ‘unspecified’ social support, including advice, encouragement, or coaching, was also noted as a promising BCT. This could be a function of interventions being delivered by healthcare professionals (HCPs) who have additional responsibilities and thus less available time to implement the emotional social support BCT. Alternatively, emotional social support may not have been explicitly mentioned where it is perceived as a naturalised component of an HCP’s role.^41^ Many interventions provided some form of lifestyle information or education. The authors assumed that education provision would involve at least two BCTs — ‘information on health consequences’ and ‘instructions on how to perform a behaviour’.^11^

BCTs were also coded in the control groups, which mostly comprised usual care. There was some overlap between the BCTs in usual care and intervention arms, with some BCTs — for example, ‘pharmacological support’ or ‘credible source’ — considered part of usual care.

The majority of interventions were delivered face-to-face, or via telephone and face-to-face. Face-to-face delivery seemed the most acceptable mode of delivery for an older population. However, one of the significant QoL interventions was web based. Others had elements of telephone follow-up. Flexibility in delivery mode may be useful in the context of COVID-19 and emerging new ways of working.

### Strengths and limitations

This review focuses on BCTs within PCP interventions specifically for older adults (aged ≥65 years). The findings add value to the self-management approach outlined in the comprehensive personalised care model as they pertain to the specific needs of older adults who fall within the 30% of the population with long-term physical or mental health conditions (including frailty) who need targeted interventions. They can easily be adopted by health and social care professionals working with this population to enhance existing health coaching skills. The authors anticipate that review findings will be especially useful in any future implementation of personalised and anticipatory care. The focus on BCTs that improve quality of life in older age is aligned with the growing recognition that this is an important outcome for older people.

The review has limitations. All interventions used multiple BCTs, but it was not possible to assess the impact of individual BCTs on outcomes. The authors attempted to mitigate this by focusing on BCTs that occurred in all five studies with significant improvements in QoL.

The heterogeneity of interventions precluded meta-analysis, and lack of follow-up data meant the authors were unable to assess long-term effectiveness. Assessing risk of bias was also problematic as unblinding of participants and delivery staff is almost inevitable in PCP.

The authors only included randomised controlled trials in this review as they wanted to examine BCTs that had been utilised and tested for effectiveness and acceptability using the same design as their own intervention evaluation. Although this potentially missed studies, including alternative BCTs, the robust evaluation design increases confidence in review findings.

### Comparison with existing literature

The authors used the pragmatic assumptions of Brown *et al*^16^ to define promising BCTs, enabling them to focus the BCT taxonomy^11^ and identify BCTs that are most useful in PCP for older people. This is particularly important as techniques appropriate for younger adults may not be effective for older adults.^42^

### Implications for research and practice

Consistent use of the BCT taxonomy when reporting interventions in research studies would enable researchers to identify specific BCTs for use with specific target populations. More widespread inclusion of mediation analysis in RCTs of PCP interventions in older age would help inform which individual BCTs are most effective.

The review findings are especially relevant for the current English primary care context, as they can be used to inform operational implementation of the proposed PCN DES personalised and anticipatory care service specifications.
